# 20∶60∶20 - Differences in Energy Behaviour and Conservation between and within Households with Electricity Monitors

**DOI:** 10.1371/journal.pone.0092019

**Published:** 2014-03-18

**Authors:** Niamh Murtagh, Birgitta Gatersleben, David Uzzell

**Affiliations:** School of Psychology, University of Surrey, Guildford, Surrey, United Kingdom; University of Bath, United Kingdom

## Abstract

The introduction of electricity monitors (in-home displays; IHDs), which show accurate and up-to-the-minute energy usage, is expected to lead to reduction in consumption. Studies of feedback on domestic electricity use have generally supported this view. However, such studies also demonstrate wide variation between households. Examining the heterogeneity of responses is essential for understanding the actual and potential effectiveness of IHDs and in order to target interventions effectively. To explore differences between households' responses to IHDs, we conducted a qualitative study with 21 households who had an IHD for more than six months. Of the 21, only four households continued to refer to the IHD and the findings suggest that attempts to reduce energy consumption were situated in wider social and physical contexts. Further, the participants demonstrated energy saving behaviour before and outside of IHD usage. The patterns of energy behaviours and attempts at electricity conservation could best be understood by categorising the households into three types: the Monitor Enthusiasts (20%), the Aspiring Energy Savers (60%) and the Energy Non-Engaged (20%). The factors of importance in energy behaviour differed between the categories. Financial savings contributed to efforts to reduce energy use but only up to boundaries which varied considerably between households. Social practices and social relationships appeared to constrain what actions households were prepared to undertake, illuminating aspects of inter-household variation. Within the household, all energy users were not equal and we found that women were particularly influential on energy use through their primary responsibility for domestic labour on behalf of the household. The implications of the findings for environmental campaigning are discussed.

## Introduction

Householders can find it difficult to reduce their domestic electricity consumption because electricity use is not only effectively invisible [1] but is also billed in aggregate, at long intervals and retrospectively [2]. The expectation follows that the provision of in-home displays (IHDs) – providing feedback of usage that is accurate, up-to-the-minute and often disaggregated by appliance - will lead to more efficient energy usage and overall reduction in consumption [2]–[4]. Reviews of studies of feedback on domestic electricity use have generally provided positive support for this view. However, we argue that this positive support has been based on aggregated outcomes, and in particular on average reductions, and that this obscures the empirical evidence for a range of responses, negative and positive, to IHDs. We aimed to examine in detail household responses to IHDs. We present findings of a qualitative study which demonstrated rather more complex and nuanced responses to the introduction of an IHD in the home, wide variation between households and important differences within households. Investigating the variety of responses is essential to understanding why householders attempt to cut their energy use and why they do not, so that assumptions about the impact of IHDs become more realistic, and environmental campaigning and policy can address the broad agenda of energy conservation with additional focus. We use the term in-home display (IHD) in preference to terms such as smart energy monitor as our focus was the impact of real-time information display, rather than any ‘smart’ functionality which is available in some but not all monitor devices at the present time.

As a background to the research, we begin by outlining the importance of IHDs in policy. We will then examine the research literature on what is known about how households respond to feedback and identify the gaps that our study aimed to address.

### Importance of IHDs: energy conservation and energy efficiency

In 2009, across the 27 European Union member states, domestic premises were responsible for 27% of the total energy consumption [5] with earlier estimates for the US at close to 40% [6]. Of UK carbon emissions in 2012, 15% were attributable to residences, in addition to a proportion of the 40% contributed by the energy sector [7]. Electricity consumption by households has increased by 39% since 1990 [5] and energy efficiency improvements in appliances such as freezers and light bulbs has been overtaken by increased use of consumer electronics [8]. The residential sector is therefore an important target in seeking to reduce overall energy consumption, in order to cut CO_2_ emissions and to establish sustainable energy systems into the future. Households are seen as one of the most promising domains for reducing emissions [9] with an expectation that changing behaviour in the home will be relatively easy to accomplish [10]. Provision of IHDs have been mandated for member states of the European Union and accurate, real-time displays are deemed essential in order to allow end-users to take better-informed decisions on their energy use, in conjunction with information on energy saving and enhanced billing [11]. Based on the assumption that better information means changed behaviour (the ‘information deficit model’ [12]), IHDs are perceived to be crucial to demand response (i.e. “actions which can be taken at the customer side of the electricity meter in response to particular conditions within the electricity system” [13]. Coupled with the potential for smart meters to enable near-real-time consumption monitoring and automated demand side management, IHDs are seen as an essential component of future energy efficient systems, enabling behaviour change in the home, leading to reduced CO_2_ emissions and electricity consumption. Demand response includes shifting consumption away from peak periods, dynamic response to market conditions and reduction in overall consumption. Because reduction of energy consumption can lead directly to reduced greenhouse gas emissions, in this paper we focus on reduction in overall consumption.

### Main Reviews of Effectiveness of Feedback

The assumption that IHDs will precipitate behaviour change is based in part on a number of reports and reviews of field studies from the UK, Europe and the US, most of which have found positive outcomes for in-home feedback [14]–[17]. Although overall conclusions are generally positive, the details show a more mixed picture. For example, Bittle et al. [18], Brandon and Lewis [19] and van Houwelingen and van Raaij [20] found that medium and/or low consumers increased their energy use during trials. In the Brandon and Lewis study [19], only one intervention type showed a statistically significant number of households in which energy use decreased: for the other six intervention types, almost equal numbers of households increased as decreased. Faruqui and Sergici [21] noted that 80% of the improvement in demand response came from 30% of the participants. One of the AECOM studies found significant reductions of up to 1.5% in three out of six trials but increases of up to 14% in the other three [16]. Indeed, where studies have been able to examine access to feedback, the lack of engagement by a substantial proportion of participants has been evident: 40% [22], [23] to 50% [24] of participants did not access their feedback. In attempting to interpret the findings of often complex combinations of trial conditions, both within studies [16], [19] and within reviews [14], [15], [17], researchers have tended to use aggregated findings in order to produce an estimate of the general effect. In particular, the average reduction in energy consumption is typically calculated: an understandable focus given a primary objective of feedback being to reduce consumption. However, such an approach suffers from the weakness of obscuring overall patterns of response and variation between responses. While useful in depicting the total outcome of the trial sample, it hides what is happening at a unit level, that is, by household. We suggest that the aggregation of outcomes to calculate the mean change across participant households is obscuring a pattern of wide variation. Critically, understanding such variation is necessary in order to target interventions most cost-effectively and essential to determining the effectiveness of IHDs and the potential for overall reduction in consumption [25]. The current study aimed to examine household responses to IHDs in detail, to illuminate heterogeneity in responses and thus to provide greater accuracy in understanding the potential effectiveness of IHDs.

In examining the variation in how individuals and households understand, experience and react to their environment, qualitative research methods appear particularly appropriate. Previous qualitative studies have contributed to knowledge of household energy use and responses to IHDs and salient findings are now briefly outlined. Although the cost of energy has relevance, activities in the home have symbolic value so, for example, constructing cosiness and comfort may be more important than expense in determining levels of energy use [26]. More generally, the meaning of activities in the home are culturally influenced [27]. Motivations for engaging with the IHD may be mixed and include the environmental (carbon reduction), technical (interest in gadgets) and financial (money saving), although the small savings realised by changing behaviour may be perceived as frustrating financial motivation [28]. In their UK-based study with 15 households, Hargreaves and colleagues [29] found that the IHD devices in their study appeared to have gendered appeal and to have one main user, usually a male. In addition to observing the negotiations generated within the household by the IHD, their insightful study noted quite dramatic variation in energy use characterised as necessity, ranging from comfort and warmth to fish tanks. Their participants indicated that they became less engaged with the IHD over time, and this echoed quantitative findings that initial gains in energy conservation after introduction of feedback were not maintained over the longer term [29]. A follow-up study 12 months later with 11 of the original households emphasised the attenuation of impact of the IHD with time [30] and this was the first study to our knowledge which explored qualitatively temporal patterns in responses to IHDs.

Beyond these useful insights, gaps remain. Although several writers have argued that domestic energy use, and efforts at reduction, must be seen within a broader context [28], [31], there have been few attempts to explore the broader social and physical landscapes in which households make energy-relevant decisions. Domestic behaviour (and thus domestic energy use) and behaviour change may not fall equally to all household members [32] but many studies on domestic energy feedback, and economic models of domestic energy use, tend to treat the household as a single unit: differences within households remain underexplored. It has been argued that people do not consume energy per se, rather they use culturally meaningful services [33] and energy use has been described as implicit in practices and routines [27]. A potential benefit of IHDs is to render energy consumption more visible [1] but how does this fit with or disrupt customary cultural practices?

To examine in detail household responses to IHDs, and with a particular interest in illuminating heterogeneity, we chose a qualitative methodology. A quantitative approach would have necessitated a priori determination of the factors of interest, and pragmatically this limits the extent to which context can be explored. In contrast, the choice of a qualitative approach permits identification of novel factors, from the accounts and insights of energy users themselves, and exploration of context, both macro (such as participation in a community project) and micro (such as relationships within the household). Quantitative analysis typically seeks large sample sizes so that findings may be generalised. In contrast, although qualitative methodologies must work with small samples, they offer theoretical generalisability, that is, a finding which applies for one participant may in theory potentially be true for others. Qualitative analysis cannot claim to what extent such a finding may apply more generally. Nevertheless, because qualitative methods permit a detailed focus on the experiences, behaviours and practices of individual households, rather than describing overall social patterns, a qualitative approach was chosen as the more appropriate to address our research questions. The current study recruited from three different socio-geographic contexts to explore to what extent wider, macro-social and physical contexts have influenced uptake of IHDs in households. We chose households that were not part of a research trial. Although this meant that actual energy usage was not measured as access to usage records would require prior permission from participants, recent research has shown that participation in a trial, in itself, can influence energy consumption via heightened energy awareness [34]. In contrast to most existing domestic energy studies, we sought to avoid this confounding factor. We explored differences within households and how the IHD had influenced routine energy behaviours over time. In summary, the research sought to explore the experience of households who had IHDs for more than 6 months, to understand how wider contexts influenced a decision to procure an IHD, the differences between households of responses to IHDs, differences within households and energy behaviours after the ‘honeymoon’ period.

Following best practice for qualitative research, we did not approach our study with a specific theoretical model in mind as this can bias analysis and interpretation. However, the methodology applied thematic analysis [35] and drew on a number of the major theoretical perspectives which have been employed in investigating energy behaviour. The theories were drawn from both psychology and sociology. Although they emerge from different epistemological perspectives, in the absence of an adequate over-arching theory of energy consumption, we felt it appropriate to harness theories that can illuminate aspects of the processes of energy use, although as we show below, there are limitations to each theoretical strand. Space does not permit extensive consideration of the theories: a thumbnail outline is given and the interested reader is referred to the reference sources. Social practice theory [36] focuses on routine, everyday sets of actions or ‘practices’. Practices emerge and develop within socio-cultural contexts and are understood through common meanings and performances, and often common technologies. Bathing and washing clothes are examples of social practices, and sustainable behaviour is seen as embedded within practices [37]. The extensively researched theory of planned behaviour (TPB) [38] proposes that individual attitudes influence action although there is also robust evidence of the disjunction of attitude and behaviour [39]. Values, the relatively stable and context-independent guiding principles of individuals' lives, are argued to direct behaviour, and biospheric or ‘green’ values may be of particular salience in environmentally-impacting action [40], [41]. Finally, the self-determination theory of motivation [47] proposes that more internalised or intrinsic motivation supports persistence in behaviour whereas extrinsic motivation, such as financial reward, may undermine internalised motivation.

## Methods

The study design was guided by criteria for validity [42] and reporting [43] of qualitative research. In order to include households in different social and geographical contexts, we recruited in three tranches. The first set of households, the ‘Eco’ group, resided in a small housing development in the suburban outskirts of a large town in south-west England. The development consisted of a mix of one-household ‘eco-homes’, built to the highest environmental standard in the UK at the time (Code for Sustainable Homes Level 5). The occupants had been in residence for about one year and the IHD was ‘built in’ as part of the eco-systems in the homes. The second set of households, the ‘Rural’ group, was in a rural location in south England. The IHD had been provided as part of a community sustainability project. The IHDs had been installed about 9 months before the interviews and the householders had opted to have the device installed. The third set of households, the ‘Suburban’ group, were recruited by a market research agency from London and its suburbs on the basis of having an IHD, either bought by themselves or provided by their electricity supplier at least six months previously. The first two groups were selected as settings in which wider social or structural sustainability initiatives were taking place. The third group, in contrast, were not part of any ‘green’ programme. None of the participant households had had formal training on the IHD.

For the Eco group, recruitment and interviews were conducted in conjunction with a post-occupation evaluation (POE) of the housing stock, which preceded the interview for the current study. An invitation letter and a follow-up were sent to all 12 homes in the development. Three households agreed to be interviewed. The post-occupancy assessment included provision of graphs on energy usage per home but no additional incentive was provided. In the Rural group, of the 15 households with energy monitors, 12 were approached (the remaining three were either involved in running the project or lived in a different village from the main project) and 10 agreed to participate. An introductory invitation letter was followed up with a phone call. An incentive of £15 (€18) per interview was offered. Because of the small number of households in the first two groups, all households who accepted the invitation were interviewed. For the third group, purposive sampling was used to match particular socio-demographic criteria. Specifically, we recruited to ensure that participants were a mix of sole occupants, families with young children and families with teenagers. The sample provided a mix of socio-demographic factors, including income, ethnicity, life-stage and location, comparable to previous studies [28], [30]. An incentive of £25 (€30) per interview was offered (due to the higher cost of living in London) and eight households were interviewed, bringing the total number of participant households to 21.

### Participants

Table 1 summarises the participant households and the household members interviewed. Ethnicity was not a recruitment criterion and the ethnicity of participants reflected the locale: all participants in the rural setting were White British but ethnicities in the suburban households were more varied, in line with regional diversity. As the Suburban group was offered a higher financial incentive to participate, it is possible that this group had more materialistic values than the other groups. The household incomes of the Suburban group were also noticeably higher than the Rural group. This could relate to valuing material assets or could reflect the cost of living and demographics in the London area, the wealthiest region in the UK in terms of gross domestic income [44].

**Table 1:** Summary of Participant Households and Interviewees.

Ref	Number & Gender of Participants	Occupants' Ages (years)	Ethnicity	Net Household Income	Type of Home	IHD Origin & Period of Possession	Household Category
E1	1 (M)	**40**, 35^a^, 3,1	W/WB, A/AB	>£60 K^b^	D	Given >12 months	Couple with children under 10
E2	2 (F, M)	**40 s, 30**, c	W/WB	£20 k–£30 k^b^	S-D	Given >12 m	Couple with teenage children
E3	1 (M)	**50 s**, 50 s, 19	W/WB	£20 k–£30 k^b^	S-D	Given >12 m	Couple with teenage children
R1	2 (F, M)	**28, 29**	W/WB	£30 k–£40 k	D	Given >6 m	Couple without children at home
R2	1 (M)	**49,** 46, 19, 16	W/WB	£20 k–£30 k	D	Given >6 m	Couple with teenage children
R3	2 (F, F)	57, **55**, 17, **15**	W/WB	<£10 k	D	Given >6 m	Couple with teenage children
R4	1 (F)	64, **63**	W/WB	£20 k–£30 k	D	Given >6 m	Retired couple
R5	1 (M)	**56**, 55	W/WB	>£60 k	D	Given >6 m	Couple without children at home
R6	1 (F)	67, **60**	W/WB	<£10 k	D	Given >6 m	Retired couple
R7	2 (F,M)	**54, 54**, 75	W/WB	£10 k–£20 k	D	Given >6 m	Couple without children at home
R8	1 (F)	**77**	W/WB	£10 k–£20 k	S-D	Given >6 m	Sole occupant
R9	2 (F, M)	**86, 75**	W/WB	£20 k–£30 k^b^	D	Given >6 m	Retired couple
R10	2 (F, M)	**70, 69**	W/WB	£20 k–£30 k	S-D	Given >6 m	Retired couple
S1	2 (M, M)	80 s, **50 s, 50 s**, 30 s	A/AB	£20 k–£30 k	T	Bought >12 m	Couple without children at home
S2	2 (F, M)	**46, 43,** 20^d^, 17, 12	A/AB	£50 k–£60 k	S-D	Given <6 m	Couple with teenage children
S3	1 (F)	41, **40**, 9, 5	B/BB	£30 k–£40 k	F	Bought >6 m	Couple with children under 10
S4	2 (F, M)	**50, 49**, 22^d^, 20^d^, 16	A/AB	>£60 k	D	Given >12 m	Couple with teenage children
S5	1 (M)	**43**, 30, 3 months	W/WB	>£60 k	S-D	Given <12 m	Couple with children under 10
S6	1 (F)	**39**, 39, 3, 1	W/WB	>£60 k	S-D	Given >12 m	Couple with children under 10
S7	2 (F, M)	**29, 29**, 2 months	W/WB	£30 k–£40 k	T	Given >12 m	Couple with children under 10
S8	1 (M)	**39**	W/WB	<£10 K	S-D	Given 3 m	Sole occupant

Key.

Ref: E =  Eco group; R =  Rural group; S =  Suburban group.

Gender: F =  Female, M =  Male.

Occupants' Ages: **bold** indicates household members interviewed.

Ethnicity:W/WB = White/White British; A/AB = Asian/Asian British; B/BB  =  Black/Black British.

Net Household Income: Median household income for England in 2011 was £22,000 (€26,000).

Type of Home: D =  Detached; S-D =  Semi-detached; T =  Terraced; F =  Flat.

IHD Origin & Period of Possession: Self-bought or given by utility/project; time in months.

a Plus two70-year-old relatives for 3 months each year.

b Estimated.

c Plus 14-year-old every other weekend and school holidays.

d At university in term-time.

### Procedure

The University of Surrey Ethics Committee reviewed and approved the study. Participants signed an Informed Consent agreement and, for the case where a minor participated, the parent signed on her behalf. The interviews took place in the participants' homes and were conducted with one or two family members, as indicated in Table 1. All participants were invited to include partners and children, and the number of interviewees was the decision of the participants. Each interview took approximately one hour. All were conducted by the lead author and were audio-recorded and transcribed verbatim. The interviewer was a researcher in environmental psychology and experienced in qualitative research. In preparation for the research, she had installed an IHD in her own home, in order to observe its influence on family behaviour and interactions and to allow greater insight into the experiences of the participants. The interview was semi-structured and comprised questions on the topics of: reasons for and influences on procuring the IHD; barriers to reducing electricity consumption; differences in energy use and conservation within the household. The type of IHD varied between the participant groups and within the Suburban group. Feedback in any form may influence behaviour [15] and as the research focus here was the social-psychological aspects of household experience and behaviour in response to electricity use feedback, details of the type and functionality of the IHD were not examined. The topics were addressed through general questions first, followed by more specific follow-up where necessary (see Supplementary Information S1 for the interview schedule). There were additional questions on demand response at the end of the interviews: these are the subject of a separate paper and are not described further here.

### Analytic Procedure

Thematic analysis was chosen as the analytic method because it does not assume a specific theoretical perspective. It is an appropriate method for a realist epistemology (necessary, we believe, for a domain such as energy behaviour), allowing the recognition and analysis of specific behaviours, but also facilitating the harnessing of theoretical perspectives to explore latent themes. The methodological guidelines of Braun and Clarke [35] were adhered to. All transcripts were first cross-checked with the audio recording. After familiarisation with the data through repeated reading of the transcripts, guided by the research questions and the analytic approach, codes were identified by tagging relevant textual segments. We aimed to ensure that each data item (sentence) received equal attention and we were particularly attentive to prevalent as well as contradictory statements. Next, themes and subthemes were developed by systematically aggregating coded segments, which were conceptually similar. Moving backwards and forwards iteratively, the themes and codes were refined as analysis proceeded and in this process, theoretical perspectives from the literature were drawn on to interpret latent themes. The analysis was assisted by the use of computer software to organise codes and notes (MAXQDA 10). An initial write-up was used to construct a narrative account of the thematic map, with extensive use of extracts. An unusual and unplanned aspect of the analysis was the decision to categorise participants into three groups. This strategy allowed the clearest account of the data, which reflected the detail and complexity across all participants. In order to assess the validity of the interpretation and whether the analysis was supported by the data, the narrative was presented to a group of four researchers with relevant experience. Some minor improvements were suggested and have been incorporated into the account below.

The criteria for conducting and analysing qualitative research (e.g., ‘reliability’ and ‘validity’) have to be replaced by different but no less demanding standards than those employed in quantitative research. Yardley [42] suggests the use of four broad principles: sensitivity to context; commitment and rigour; transparency and coherence; and impact and importance. The present study has sought to conform to these requirements, and we have interpreted them in the following ways: s*ensitivity to context* requires an awareness of the milieu and a rapport-building and empathetic interview style; *commitment and rigour* requires thorough and systematic attention to the participants' accounts and analyses that strive to be honest, accurate and complete; *transparency and coherence* refers to a thorough explanation of all research steps to both the participants and the ultimate reader; *impact and importance* strives to produce outputs which are meaningful.

### Findings

In the extracts below, the group (E Eco, R Rural, S Suburban), interview number and gender of the speaker (f female, m male) are identified, for example, R10m is the 10^th^ interview in the Rural group, male speaking. Our initial assumption was that, compared to the general population, the participants would represent a relatively engaged sample with respect to energy saving, because they had purchased an eco-house, taken part in a community sustainability project or simply acquired an electricity monitor. However, our assumptions were not wholly borne out:

We have enough money to not bother [saving energy], which sounds awful, doesn't it? [R10m].

But I think to stand up, the way that most of these guys stand up and they start claiming global warming, they should rather keep that crap to themselves. Excuse my French! Because some people believe in it, some people don't. I don't believe in it, right. [E3m].

Although our sample may have been more engaged than others in the general population, they appeared to spread along the spectrum of engagement.

### Engagement with the IHD and Energy Conservation

Earlier research had suggested that active use of the IHD was unlikely more than six months after installation [29], [30], and this was reflected in our sample. At the time of the interviews (that is, at least six months after procuring the IHD), of the sample of 21 households, 17 were not using the monitor. Six of these households had never used their IHD, three of which had technical problems. A further eleven households were no longer using their display and their responses accorded with the earlier findings that the monitor was novel initially but interest then declined. Only four households in the sample continued to make active use of their monitor. However, from an energy conservation perspective, the picture was not as bleak as it may appear. In addition to these four households, a further five enacted extensive electricity-saving behaviour (as discussed below) and most of the remainder of the sample also tried to save electricity to some extent. Clearly, the IHD enabled energy reduction for some households but energy saving behaviours were being pursued more widely and without recourse to the monitors.

Sources of information and knowledge over and above that provided by the electricity monitor appeared to influence behaviour. Three households used their existing electricity meter to see whether usage was high or to take readings and plot usage graphs themselves. A further two households accessed this type of information on the website of their electricity provider and could view and compare their annual usage. Some participants felt they knew the relative consumption of their appliance through appliance rating and similar information:

For instance, we'd bought a new freezer, then we'd look at the energy consumption, and when you get the goods delivered, you get an instruction leaflet and it has all the information in the back there about how much it uses [R4f].

Others believed they were aware of appliance consumption without the monitor. Although the IHD could provide accurate, quantitative data on actual consumption of appliances in the home, some participants appeared to find their existing knowledge adequate.

As a specific example of drawing on wider sources of information, several participants mentioned that they were aware of the tumble dryer as a heavy user of electricity: “But, you know, everyone goes ‘Tumble dryer – don't use it! Don't use it!” [S3f]. Thus participants were integrating a number of sources of information to inform their beliefs on electricity usage: sources from outside the home, from the ‘marketplace’ in the case of new appliances, from utility companies' information, from other people and elsewhere. IHDs were only one means of providing information on electricity and the unique level of accuracy, personalisation and detail offered by the monitors was not universally valued. Their usage of the information provided by the monitor (or lack thereof) was interpreted by householders within the framework of their existing knowledge and this framework was constructed not only from the market and policy landscapes noted by Hargreaves et al. [30] but also more generally from social (and therefore presumably media) sources.

### Reasons for Adopting an IHD and for Saving Energy

Amongst the group, there were varying and multiple reasons given for adopting an IHD and/or trying to save energy. The environmental credentials of the eco-houses were a primary concern for two of the Eco group although lower spending and location were also important. In the Rural group, six participants wanted to support the sustainability project as a local initiative. Five of the Rural group and four of the Suburban group were interested in what the IHD could tell them. Across all households, 11 spoke of savings as a motivating factor (2 Eco, 3 Rural, 6 Suburban) and 11 also referred to environmental impact (2 Eco, 3 Rural, 6 Suburban): six of these households discussed both savings and environmental motivations. Thus the pattern of reasons showed an influence of the socio-geographic settings, with the social context of a community project and the physical context of an eco-house providing impetus to install or use IHDs. But the motivations were complex, with multiple reasons influencing the decision.

When it came to utilisation of the electricity monitors, and engagement in energy conservation, wide variation was apparent between households and this did not align neatly with the three participant groups. To represent and understand this wide variation, we found it useful to categorise the sample into three types of user. This allowed the analysis to examine patterns and similarities within each category and differences between categories. The categories identified were: the Monitor Enthusiasts (4 households); the Aspiring Energy Savers (13 households) and the Energy Non-active (4 households): an approximately 20∶60∶20 split.

### The Monitor Enthusiasts

The four Monitor Enthusiasts (E2, R8, S3, S6) “loved” their monitor, had it positioned so that they could refer to it frequently (on the kitchen work surface, by the TV, by the computer, in the lounge) and were very familiar with the content of its display depending on the household behaviours: “I know if I turn my kettle on that it'll go up to 66p!” [S6f]. Two participants used the display in money units, one used the kWh display and one used the ‘traffic light’ display. In the accounts of all four, money savings were frequently referred to and this appeared to be a major motivation. Saving electricity meant saving money – the two concepts were interchangeable and inextricably linked: “I see my pounds disappearing on that meter” [E2f]. Two of the four had a household income at or below the national median. However, one had an income one band above the median and the fourth household was in the top income band. In addition, three of the four referred to their interest in environmental matters when discussing their energy saving, so it can be suggested that their motivation was not only financial.

In the accounts of the four Enthusiasts, a common pattern could be seen of how they had actively changed their behaviour over time, building their awareness and knowledge. They had become familiar with their energy-hungry practices and they had plans in place for upgrading to more energy-efficient appliances in the future. A striking note in these accounts was the effort, thought and time that the participants had put into energy conservation. For two (S3, S6), their pursuit of energy efficiency had aspects of personal goals [45] or personal projects [46], suggesting internalised motivation [47]. Both of these participants were full-time mothers and they linked their energy conservation with contributing towards the home. They viewed their efforts as making a financial contribution, important for them as they were not earning an income, and taking responsibility for this domain: “I'm the one that does the sort of switching and the changing [of energy supplier] and drives things more, I do all those types of things.” [S3f]. They appeared to derive a sense of self-efficacy from their efforts, to feel empowered and in control. This suggests that their motivation had become internalised and possibly integrated with the perception of self [47]. Self-determination theory predicts that internally motivated behaviour is more likely to persist and to overcome challenges than behaviour motivated by extrinsic factors.

Across the four Enthusiast households, there was explicit and implicit reference to the resources available to them, resources perhaps essential for the active pursuit of energy conservation which they were undertaking. They had time to research their electricity consumption: in addition to the two full-time home-makers, the third Enthusiast household was shift-working and the fourth participant was retired. The participants were aware that they had control over their time and flexibility in behaviour as a result of this: “I'm a stay-at-home mum. I've got quite a bit of time – well, around the kids!” [S6f]. The participants were not asked about educational qualifications but it was evident that they had the intellectual resources to investigate the questions on energy use they wanted to answer, to compare suppliers and to grapple with the relative consumption of different household appliances. The Enthusiasts were able to deploy time, cognitive ability, energy, interest and motivation in their pursuit of energy conservation.

### The Aspiring Energy Savers

We included the majority of the sample in this category. All expressed interest in saving energy or concern over how much they were using although these households varied considerably in the actions they undertook to save electricity. Almost half of the participants in this group were interested in the information on energy use the IHD could provide but a dominant theme in most accounts was money. As with the Monitor Enthusiasts, energy and money appeared to be interchangeable:

And I do tell them…’If you're not using the room lights…that little bit of nagging has probably saved me another couple of hundred quid! [S2m].

For the majority of participants, all savings were seen as important:

Big things are only made of small things, that's the way I look at life [R2m].

But it all mounts up in the end, doesn't it? [S4f].

It's addition: you know, 60 watts, 100 watts on one bulb is nothing, but it adds up [S1m].

This contrasts with Hargreaves et al. [28] and others who have found that the money saved by conserving electricity may be deemed too small to be worth pursuing. However, energy prices and the cost of living in the UK have climbed steeply in the last three years and the findings here may indicate greater cost-sensitivity of households in the current economic climate. There were indications that, for some, what is important was not so much the monetary value or money equivalent but the fact of saving. This made sense of the emphasis on savings in households near or at the highest income range. For other participants, saving electricity was a way of living:

Both of us were already in the habit… it was always switch the lights off, don't leave that plugged in, you know. So, again, it's kind of always been a way of, a way of life, to be honest. [R2m].

Some attributed this to their childhood or previous experience. The theme of making savings, not only on electricity but wherever possible, recurred in their accounts and appeared to influence many of their actions. For these participants, it can be suggested that not wasting electricity was a value, a guiding principle in how they lived. Previous research has demonstrated the effect of biospheric or ‘green’ values on environmentally friendly behaviour [48] and the analysis here suggests that other values may also influence pro-environmental action. Although half of our participants mentioned environmental impact as a factor of importance to them, this did not wholly align with saving electricity as a way of life: some habitual savers (including R2m quoted above) did not mention environmental impact. This speaks to the argument of Evans [49] who differentiated between thrift (saving to spend elsewhere without reference to reducing environmental impact) and frugality (saving as a moral imperative as part of a pro-environmental agenda). In the current sample, households which could be deemed either ‘thrifty’ or ‘frugal’ attempted to reduce energy consumption. The analysis thus suggests that values other than biospheric may also encourage domestic energy conservation.

Despite emphasising their motivation to save electricity, when asked how they could cut their energy consumption, almost all in this group were able to identify behaviours that could potentially be changed: from low consumption actions (e.g. unplugging a mobile charger when charging had completed) to high (e.g. not using the tumble dryer when it is sunny, not using multiple TVs simultaneously). Thus, on the one hand, the participants appeared to be motivated to save energy but, on the other, to be aware of further actions they could take yet had not done. Their accounts showed complex thinking about their energy behaviours, which did not align with a rational, linear model of behaviour [50]. In particular, their responses made clear that it was not a lack of information that hindered their energy conservation.

Several participants felt that they were already conservative in their use of electricity: “We're very good… I mean, putting things into sort of standby mode, you know, I mean, we always try and do that.” [R5m]. In seeing themselves as “very good” (or “naughty” [R10m]), participants demonstrated awareness that saving electricity is considered socially desirable. Further, they positioned energy saving as a moral issue and this may be an important additional dimension in which to engage householders in energy conservation. Having constructed energy saving as socially desirable and morally right, the participants also portrayed themselves in a positive light in this regard. This may be an outcome of the acknowledged and pervasive cognitive bias towards excessively positive self-evaluation [51] and, at the least, indicates a desire to feel oneself to be as good as or better than others in this domain. Positive illusions about the self have been linked to happiness, life satisfaction and well-being [52] but, in the domain of sustainable behaviour, they may serve to protect the individual against awareness of a need to change and function to de-motivate action to change.

Moral judgements then may explain differences between households in their attempts to save energy and the limits they place on their behaviour. Participants' perceptions of what constituted ‘good’ energy conservation was highly subjective: the habit of Household R5 of putting appliances into standby mode contrasted strongly with another household's concern over the light emitting diode (LED) on the washing machine:

I'm loathe to be out when I know the cycle's going to be finished, ‘cos there's a tiny little LED on that… but it's burning a bit of electricity! [S8m].

This raises an important and difficult question in domestic energy use: to what extent is it possible to deem specific energy use wasteful? Hargreaves et al. [28] found that uses such as lava lamps and fish tanks were considered essential (and therefore not wasteful) by some participants. Who can determine what is essential or wasteful? The embedded nature of energy use makes the question more complex: *how* may we determine what is essential or wasteful? One potentially useful means to explore this question is by the contrasts that participants themselves drew between their own current and earlier behaviours. Participant S6 (now a Monitor Enthusiast) said of her earlier viewpoint:

And I just thought, ‘No, actually it's a new modern house. It's, you know, energy-efficient. We've got all, you know, A-rated appliances. We can't do anything about it. We are as best as we can get.’ [S6f].

In this extract, she appeared similar to Aspiring Energy Savers: interested in saving energy but believing her household to have achieved all they could. In part, she had depended on technology to be energy-efficient on her behalf, in both house construction and appliances, and she also suggested that changes were beyond her (and her husband's) control. The claim that they are the “best [they] can get” may be linked to energy saving as a moral good, as discussed above, and to others' claims to be “very good” in their energy use. She had made many changes as a result of her active pursuit of energy saving including extensive use of the IHD, begun when she started full-time parenting. The changes included turning off the underfloor heating in two rooms which had been on continuously for five years, less frequent use of the tumble dryer, and setting the house thermostat at 19°C rather than 22°. In retrospect then, Participant S6 reflected on the wastefulness of earlier behaviours although, at the time, those behaviours appeared to be both ‘good’ and not changeable. These latter perceptions appeared in the responses of other participants in the Aspiring Energy Savers category. Because such perceptions constrain efforts to reduce energy consumption, of particular interest was why particular energy uses were perceived as unchangeable.

Amongst behaviours seen as not possible to change were washing (“Can't really not wash” [S7m]), heating (“when you need the heating or the electricity you can't really turn things off” [S1m]) and clothes washing. Such perceptions may be understood with reference to social practice theory, which explains human behaviour in terms of practices, that is, “coordinated entities of sayings and doings” in which particular elements hold the practice together, and with collective continuity through time and space [37]. Thus there are culturally acquired and commonly recognised practices and expectations around personal hygiene, washing clothes and so on. Because the meanings are held in common and have continuity over time, individual ‘carriers’ (or performers) of a practice will see it as inevitable, not possible to change and not possible to challenge. For the participants, the energy behaviours of teenagers too were considered inevitable and energy use was positioned as implicitly embedded in social norms: “And iPods, everyone has an iPod,… it's just part of something that they've got to do, really” [S4m].

Themes around desired levels of comfort, convenience and warmth were also common: “I'd rather spend that extra £10 and keep the house warm than to sit here with three jumpers on” [S2m]. These themes have been understood by previous researchers as culturally, historically and physically situated [31] but the tension within the individual between levels of comfort and savings are evident in this extract. Here, the participants appeared aware of alternative meanings and practices they could pursue but chose not to. Social practice theory can help to explain why particular uses of energy may appear as inevitable and unchangeable, limiting efforts to save energy, but provide less insight into why individuals or individual households differ in their practices or may choose one practice rather than another.

Beyond moral judgements and social practices, there were further social factors which limited participants' ability to save electricity. Concern for the care and well-being of family members emerged in several accounts: keeping the house warm for an elderly parent, for a spouse, for visiting family and for teenage children meant higher energy use:

My son… has been revising for exams…at the end of the day you want him to be in a room where it's nice and warm and he can revise. You don't want him…getting cold and that. So we just, look, if he needs [the heating] on, just put it on. [S4m].

More generally, concern for children meant observing boundaries to conservation efforts:

See, with the kids and that, I wouldn't change anything… if it came to me sort of saying to them, ‘Oh right, well, I don't want you using your laptop…’,if it's going to upset their rhythm of work… I wouldn't want to change that. I wouldn't change that at all. [S2m].

Again, tension was in evidence in listening to the participants: the fathers in these extracts demonstrated an internal argument in which they are aware of their desire to save energy but must negotiate the conflict this generates with their desire to attend to their children's perceived needs. Earlier research found a relationship between social identities and sustainable behaviour [53]. In the extracts here, dynamic tensions within individuals were apparent as participants tried to enact their identities as caring fathers.

### The Energy Non-active

The four Energy Non-active households (E1, E3, R9, R10) were diverse in life-stage and form of housing, and included an elderly couple, an actively retired couple, a family with young children in a five-bedroom eco-house and a family with one teenager in a two-bedroom eco-house. All four households were aware of the environmental need to save electricity. Despite his non-acceptance of anthropogenic global warming, Participant E3m said:

Look, I know we need to save energy. And I know that energy's going to become more and more expensive… It's just the way everything is dependent on energy. And we need to start thinking more about energy… [They should] do it on the basis of, ‘Listen, you are going to be using more energy because of computers and modern TVs and this and that and that. Would you be interested in saving?’ Course I would! [E3m].

But this stood in contrast to his electricity consumption which was much higher than comparable houses in the eco development. Participant E3m attributed the high consumption to a “big-ass TV” (approx. 1.4 m) which he used during the day as a radio, and computers, servers and games boxes on all the time. Another participant in the Non-active group recognised the individual and collective responsibility for energy conservation: “And yet we should all make an effort, shouldn't we?” [R9f]. However, the same participant acknowledged that they were sufficiently affluent not to need to save electricity, a perception echoed by Household R10. Interestingly, the income band of these two households included the national median income, that is, they were not ‘rich’ in terms of net household income. Nevertheless, they felt they “have enough money to not bother” [R10m]. Thus wealth could be seen as a factor contributing to the lack of engagement of these two households in energy conservation. It was not the absolute value of their income, but rather their perception of their disposable income compared to their spending on energy.

As noted for the Aspiring Energy Savers group, the actions of these participants could be seen to contradict the opinions they expressed. On the one hand, these participants expressed pro-environmental attitudes and awareness but on the other, explained why these did not affect their own energy behaviours, by positioning energy conservation as only necessary (for themselves and others) if there were financial factors forcing behaviour change. Their argument proposed a need for external or extrinsic motivation to change their behaviour: “We wouldn't make great efforts unless it was seriously impinging on the pocket” [R10m]. However, such claims can be understood as emanating from a pervasive ‘rationalist’ model of the person, rooted in an economic perspective that assumes that external incentives, and especially financial incentives, are necessary (and sufficient) to engender behaviour change [50]. Self-determination theory has proposed, and empirically demonstrated, that in fact externally motivated action will only continue while adequate incentive is in place [47].

### Differences within Households: Gender

We examined differences within the household by asking who *used* more electricity and who *cared* more about electricity use, as well as noting who used the IHD. Previous research had found gender differences in use of IHDs [28], [30], [54]. Our data also showed heterogeneity within the household although at a more complex level than suggested previously.

With respect to use of the IHD, a number of households demonstrated the same gendered pattern noted in previous studies: the husband was interested in the monitor, the wife did not get involved. However, in contrast to these studies, overall we found an even split between genders of interest in, and engagement with, the monitors. When it came to caring about electricity use in the home, women were slightly more likely to care more: in 7 households, participants felt they cared equally; in 6 households, women were more concerned and in 4 household, men were the more concerned. (The four remaining households comprised two single occupants and in two households (E1, E3), the question on relative concern was not asked due to time constraints). For many, the person who cared more also paid the bills. In some cases, it was not the main wage earner who took responsibility for bill paying. There appeared to be accepted division of responsibilities within couples: one person was responsible for home administration tasks which included paying bills. Concern over electricity use tended to align with this responsibility, rather than whose income paid the bill, although of course, in some households, the main wage earner paid the bills and was most concerned about energy use.

A clear difference emerged when it came to use of electricity. Two households felt their teenagers used most, through use of laptops, mobile phones and music players. In two households, the man used most, due to use of welding equipment in work or hobby and a further three households believed that use was equal. However, in ten households, the participants felt the woman used the most, because she took primary or full responsibility for domestic chores including cooking, clothes washing and vacuuming. Thus, in carrying the major load of domestic work [55], women may be the primary consumer of electricity on behalf of the household. Other researchers have explored the impact of domestic technology on women's lives [56]–[59]: here, we make the link to energy consumption.

Further, in ten households, there was evidence of the woman's wider influence on energy use and attempts at energy saving. Consider the following exchange in a household where the husband has been more interested in the IHD and more concerned about energy use:

Man: I was going to…harangue my wife… and say hang the washing out rather than use the tumble dryer, but I haven't got round to doing that. But that would probably be one of the things, if we saw how much it was costing us… Would it have an effect on you, dear?

Woman: No.

Man: No. It would me....

Woman: I probably feel that the tumble-dryer would still outweigh the hanging it out…

Man: But we could research that now we've got the energy monitor.

Woman: Well, you could research it, and if it was a huge amount, then I would try, but if it wasn't a huge amount, just for the convenience of it, then I wouldn't. [R5]

In this extract, despite the husband's awareness of possibilities for energy saving, it is the wife who will decide what is done. Participant S3f was clear: “I'm far more influential.” This balance of power appeared to be accepted by the husbands in our sample and can be understood as operating alongside, or because of, the wives' primary contribution to domestic labour. The gender of the interviewee(s) did not appear salient to these themes: differences in caring about how much electricity was used, in use of electricity and in influencing electricity use emerged where the interviewees were men, women and couples.

## Discussion

Semi-structured interviews were conducted with 21 households to explore different responses to IHDs, between and within homes, six months or more after installation. Participants were recruited from three different socio-geographic settings in southern England: from an eco-house development, from a community sustainability project and from households not involved in an environmental initiative. Although differences in reasons for procuring and engaging with an IHD could be related to different physical and social contexts, other, more extensive differences in patterns of response to the IHD and attempts at electricity conservation were observed. Categorising the participant households into three types: the Monitor Enthusiasts (4 households), the Aspiring Energy Savers (13) and the Energy Non-Engaged (4), and by examining in detail differences between and within households, we suggest that this study offers the first detailed exploration of heterogeneity of household response to electricity feedback.

The categorisation that we found useful and appropriate for our sample fell into proportions of approximately 20∶60∶20.This is similar to that suggested by DEFRA [60], with the Monitor Enthusiasts here reflecting their Positive Greens, and the Energy Non-engaged mapping to their Honestly Disengaged. As a qualitative study with a small sample size, the current study cannot be considered statistically representative. Nevertheless, its reflection of DEFRA's environmental segmentation model suggests that our categorisation offers detailed insight into diverse responses to electricity conservation, across a spectrum of difference already proposed. We argue not only that our findings help to explain the variation found in many studies of IHD in domestic settings but that many of the positive outcomes, as measured by averages, may be attributable primarily to the Monitor Enthusiasts in each trial. Thus the findings challenge an assumption of universal influence of IHDs on energy behaviours.

While challenging an assumption of causality between IHDs and reduced energy consumption, this study also demonstrated that attempts to cut consumption in the home were situated in wider social and physical contexts. There was evidence illustrating the influence that social context, such as community projects, may have, as well as the effect of the physical environment, seen in the Eco group. The IHD provided information and enabled behaviour change for some households but overall, the participants demonstrated energy saving behaviour before and outside of monitor usage, and drew on knowledge on electricity use beyond that offered by the monitor. This raises interesting questions for further research: how has the common understanding of, for example, tumble dryers as high consumers of electricity come about? What discourses have informed people's knowledge? What other sources of information are effective? Future campaigning, policy and research should consider electricity feedback displays as one of many factors contributing to a wider set of processes by which people understand electricity use and try to reduce their consumption.

The differences between households showed the complexity of energy behaviours and of engagement with energy reduction. The factors of importance in energy behaviour for the Monitor Enthusiasts differed radically from the Energy Non-engaged. As well as awareness of the environmental impact of their electricity use, the Enthusiasts demonstrated interest, motivation and knowledge and invested time, energy and ability in actively pursuing energy conservation. We can suggest that little more than continuing provision of information to this group, as technology changes, will support ongoing efforts to reduce energy use. However, it was clear that the behaviour demonstrated by this group benefitted from an alignment of several factors, including individual psychological, lifestage and physical contextual factors, and that this alignment of positive influences is not easy to replicate for other households. Together with the evidence in their accounts of the many actions already taken to save electricity, this suggests that there may be limited additional benefit in energy conservation to be gained from this group.

The Non-engaged also demonstrated awareness of the environmental impact of electricity use and that energy should be conserved. However, a lack of impetus to act on this awareness was clear, and some of this group attributed their inaction to the lack of financial incentive. This group did not perceive a sufficient financial inducement in conserving electricity use at the present time, in contrast to many in the Aspiring Energy-savers group, and this reveals the subjective nature of pecuniary reward or punishment. The households who designated themselves as rich enough not to bother saving energy were not in the highest income bands – their perception of affluence was relative to the cost of energy, presumably in combination with other costs and priorities associated with their desired lifestyle. It is likely then that attempts to engage this group through economic incentives will be ineffective. Nonetheless, there are greater potential gains to be made than in the other groups because, by definition, this group has not yet engaged with energy conservation. The finding that some householders saw the issue of energy-saving as a moral issue, in that they saw themselves as good or bad in terms of their energy behaviours, may provide an alternative avenue to explore for behaviour change. Indeed, the thrust of some energy campaigns focuses on doing the ‘right thing’. However, such ‘policy into practice’ options from a moral perspective have tended to be restricted to educational strategies and to individual action. We suggest alternative mechanisms and perspectives below.

The qualitative approach allowed the complexity of motivations for behaviour to be considered. In particular, the findings noted competing motivations and behaviours *within individuals*: the father who wanted to save electricity but also wanted to keep his family warm, the participant who expressed interest in saving electricity but used his TV as a radio; and such nuanced details may not emerge in quantitative research. Such findings, together with the evidence that most householders were aware of additional ways they could save electricity, challenge the information deficit model on which assumptions of IHD effectiveness are based. Although information on energy usage may be useful and may in many cases be necessary, it is not sufficient to change behaviour – a finding long observed in health psychology [61].

Many households considered the financial savings associated with reducing their energy consumption so the cost of electricity appeared to be a factor influencing energy conservation. When it came to money, however, the savings appeared to serve a symbolic as well as a monetary function and for many participants, all savings however small were important. But the motivation engendered by monetary benefit was limited by complex social factors, aligning with earlier findings on energy use [28] and on consumption more generally [62]. There were radically different perceptions of what conservation actions were acceptable for each household, illustrating the difficulties of determining what constitutes wasteful energy behaviour or the ‘energy-efficiency gap’ [63]. Social practice theory provides insight in its explanation of patterns of behaviour as carrying social and cultural norms and expectations, leading to unquestioning acceptance and routine exercise of such practices: an individual will not deem an action as wasteful if it's ‘what we do’. However, the differences between households suggest the need for theoretical insights beyond social practice theory. Reflecting further on social dynamics within the home, we noted how social identities and social relationships influenced energy behaviours. For example, the accounts of some parents demonstrated a tension between their desire to save energy and their concern for family. Even without the strong social bonds of family, simply sharing a space means that energy behaviours must be negotiated. Energy use in the home is deeply socially embedded, not only in social practices but within social relationships and the enactment of social identities. The need for communion or belonging is argued to be a primary motivator of the individual [47]. As such, the nurturance of social relationships will take precedence over concerns for money or environment (so long as the other core needs for competence and autonomy are satisfied). Neither information on an electricity monitor nor higher energy tariffs will persuade the caring father to turn down the heating and risk his children suffering the cold. Understanding the socially embedded nature of domestic energy use can therefore explain in part the heterogeneity of responses to IHDs.

Our findings, which demonstrate that energy monitoring and subsequent decisions have to be understood in a social and family context, reaffirm previous research [28], [30], [64]. Care for the family, maintaining harmony in the home and enacting social identities were social factors limiting actions to conserve energy. This would thus suggest that energy-saving campaigns that focus on changing behaviours at the ‘appliance level’ need to take into account the social context of everyday practices in which people engage and the importance of energy consumption in fuelling lifestyles. It is noteworthy that when advertisers attempt to sell products and services, they do not appeal to rational argument but rather sell the ‘lifestyle benefits’ and appeal to social identities and affect: consider the many advertisements depicting the happy family in the cosy home. On the basis that ‘the Devil shouldn't have all the best tunes’, perhaps it would be more effective if environmental campaigns employed the very marketing techniques that energy-related companies use to persuade us to consume more and turned those messages around by linking reduced energy conservation with lifestyle and care for family. Connecting back to our suggestion above, we propose that more effective campaign messages and discourses could combine the concept of energy conservation as a moral issue with social context: doing the right thing for your family, being a good neighbour, being a model worker.

In the analysis above, we noted that, within the household, all energy users are not equal. Decisions made in managing the home have energy consequences, not only for the person doing the work but for the household. Thus the individual (or individuals) who takes responsibility for the “repetitive and non-discretionary” household tasks [65] is more important when it comes to energy consumption. In many homes, it remains the woman [55] who undertakes this work on behalf of the household. In our sample, the influence of women on energy use in the home was in evidence. The women we interviewed were at least as concerned as the men to save energy and some, though not all, found their IHD useful in doing so. In seeking to increase engagement of householders in energy conservation, although men have influence in the home, we argue that it is more important to address women's lives and concerns. In the development of technologies such as energy monitors, the question can be posed: to what extent are women involved in design and development? More broadly, to what extent does provision of a technical gadget emerge from and reinforce assumptions around the masculine nature of technology and energy? Feminist theories of technology note the marginalisation of women from technical development [66]. The evidence here demonstrates how such assumptions neglect the embeddedness of energy use in social practices and relationships, and in domestic routines. Questioning such assumptions may lead to a broader conceptualisation and understanding of energy use and of opportunities for energy saving and Wajcman [66] argues that women's involvement in technological innovation is “imperative” in order to ensure the ‘appropriation’ of technologies in the home.

In conclusion, a qualitative focus on the differences between and within households in their responses to IHDs over time has challenged an assumption of the universal effectiveness of IHDs in changing behaviour. Electricity monitors should be considered as only one component of encouraging energy conservation, for some households and within wider social and physical contexts. Financial savings contribute to reduction in energy use but only up to boundaries which vary radically between households. Social practices and social relationships appear to constrain what actions households and individuals are prepared to undertake. Through their primary responsibility for domestic labour on behalf of the household, in many homes women are particularly influential on energy use and their experience and concerns could usefully inform technical, policy and campaigning approaches to reducing energy consumption.

## Supporting Information

File S1 **Interview Schedule.**
(DOCX)
