# Supplementary material for: 20∶60∶20 - Differences in Energy Behaviour and Conservation between and within Households with Electricity Monitors
Source: PLoS One. 2014 Mar 18;9(3):e92019. doi: 10.1371/journal.pone.0092019 (PMC3958409; doi:10.1371/journal.pone.0092019)
Supplement: File S1 — Interview Schedule. (DOCX) [file pone.0092019.s001.docx]

# Supplementary Information S1 Interview Schedule

***Introduction***

[Topic of research: using electricity at home and energy feedback.

Format and duration of research.

Assurance of confidentiality, anonymity and right to withdraw.

Permission to audio record.

Request to sign Informed Consent. ]

May I start with a few details about your home? If there is any question you’d rather not answer, that’s fine.

Demographics

- How many **people** live in this house?
- Could you give me approximate **ages** (nearest 5 years) for adults? Ages of children?
- Which letter describes your **ethnicity**?
- Which letter is the range of net household **income**?

House

- This is a semi-d/terraced/detached/maisonette/flat?
- What form of heating have you got? Thermostat centrally or on each radiator?
- What form of water heating have you got?
- When did you get your electricity monitor?
- Do you have solar panels? For heating hot water or for generating electricity?

***General Reaction***

The reason we approached you is because you have an electricity monitor which [was part of the eco systems of your house/ provided by the community sustainability project/ bought for yourself]. Does this sound right?

How long ago did you get it?

Have you found it useful at all?

[Do you think it has helped you to save energy or money?

How have you been getting on with your electricity monitor?

How often do you check it?

Has 1 person used it more than others?

Has your use changed over time?

***Reasons***

Can you remember why you decided to buy the monitor?

*Social nature of implementation – external*

Did you know any other people who had bought one?

Was that part of your decision?

*Internal*

Were you (all) equally in favour?

Was negotiation necessary?

[How did that go? What did you negotiate/disagree on?]

[Reasons not to use: You said that you don’t use it (any more). Why is that?]

***Barriers***

It’s not easy to make big changes to how we use energy. Do you think you’ve changed your electricity use in any way?

[How?]

Have you had any difficulties?

[Were there ways you live that limited what you could do? Habits or routines?]

If you really had to use a lot less electricity in this house, what would have to change?

***Social Embeddedness (Intra-Household)***

Now, changing the topic a bit… in families/households, often different people use electricity by different amounts.

Couple: Which of you would you say *uses* the most electricity in the house?

Now who would you say in this house *cares* most about how much energy is used?

Why is that?

How much discussion has gone on about electricity use?

Is this because of the monitoring?

***Smart Grid Scenarios***

***[To be described elsewhere]***

[Thanks and close]
